# [^18^F]-FDHT PET for the Imaging of Androgen Receptor in Prostate and Breast Cancer: A Systematic Review

**DOI:** 10.3390/diagnostics13152613

**Published:** 2023-08-07

**Authors:** Luca Filippi, Luca Urso, Orazio Schillaci, Laura Evangelista

**Affiliations:** 1 Nuclear Medicine Unit, “Santa Maria Goretti” Hospital, Via Antonio Canova, 04100 Latina, Italy; 2 Department of Nuclear Medicine—PET/CT Center, S. Maria della Misericordia Hospital, 45100 Rovigo, Italy; luca.urso@unife.it; 3 Department of Biomedicine and Prevention, University Tor Vergata, Viale Oxford 81, 00133 Rome, Italy; orazio.schillaci@uniroma2.it; 4 Department of Biomedical Sciences, Humanitas University, Via Rita Levi Montalcini 4, Pieve Emanuele, 20072 Milan, Italy; laura.evangelista@unipd.it; 5 IRCCS Humanitas Research Hospital, Via Manzoni 56, Rozzano, 20089 Milan, Italy

**Keywords:** molecular imaging, PET/CT, prostate cancer, breast cancer, dihydrotestosterone, targeted therapy, personalized medicine

## Abstract

The aim of this systematic review is to provide a comprehensive overview of the role of fluoro-5α-dihydrotestosterone ([^18^F]-FDHT) for the in vivo imaging of androgen receptors (AR) through positron emission tomography (PET) in metastatic breast (mBC) and metastatic castration-resistant prostate cancer (mCRPC). Relevant studies published from 2013 up to May 2023 were selected by searching Scopus, PubMed and Web of Science. The selected imaging studies were analyzed using a modified version of the critical Appraisal Skills Programme (CASP). Eleven studies encompassing 321 patients were selected. Seven of the eleven selected papers included 266 subjects (82.2%) affected by mCRPC, while four encompassed 55 (17.2%) patients affected by mBC. [^18^F]-FDHT PET showed a satisfying test/retest reproducibility, and when compared to a histochemical analysis, it provided encouraging results for in vivo AR quantification both in mCRPC and mBC. [^18^F]-FDHT PET had a prognostic relevance in mCRPC patients submitted to AR-targeted therapy, while a clear association between [^18^F]-FDHT uptake and the bicalutamide response was not observed in women affected by AR-positive mBC. Further studies are needed to better define the role of [^18^F]-FDHT PET, alone or in combination with other tracers (i.e., [^18^F]-FDG/[^18^F]-FES), for patients’ selection and monitoring during AR-targeted therapy, especially in the case of mBC.

## 1. Introduction

In spite of many advances in diagnosis and therapy, the overall burden of cancer incidence and mortality is still high and, most importantly, is expected to further increase in the next few years [1]. In this respect, prostate cancer (PC) and breast cancer (BC) are among the most commonly diagnosed malignancies worldwide and represent unique clinicopathological entities, owing their specific biological characteristics, gene expression profiling, signaling pathways, subtypes and tumor-associated microenvironments. However, PC and BC share a strong dependence on the activity of endogenous and exogenous hormones [2]. In this respect, androgens, which are expressed differently in men and women, play a significant role in regulating tumor growth and proliferation in both of these malignancies [3].

The action of androgens, specifically testosterone and dihydrotestosterone (DHT), is mediated by the androgen receptor (AR), a ligand-dependent nuclear transcription factor and member of the steroid hormone nuclear receptor superfamily, which encompasses the estrogen receptor (ER), progesterone receptor (PR), glucocorticoid receptor (GR) and mineralocorticoid receptor (MR) [4]. AR is codified by a single copy gene, is more than 90 kb in length, is located on the X-chromosome and consists of a protein 918 amino acids in length, with three main domains: the N-terminal transcriptional regulation domain, the DNA-binding domain (DBD) and the ligand-binding domain [5]. The DBD, the most conserved domain across the many steroid hormone receptor superfamilies, is made up of two zinc fingers that can recognize and bind particular DNA sequences. Androgens bind to AR in the cytoplasm, determining a conformational change in the receptor and its dissociation from chaperone proteins; subsequently, the complex androgen/AR translocates to the nucleus, where it can bind to DNA and modulate gene transcription. Aside from this genomic AR signaling, non-DNA-binding-dependent actions of the AR have been described, originating at the cell membrane and employing cyclic adenosine monophosphate (cAMP) as a second messenger [6].

It is worth mentioning that PC exhibits a strict dependence on AR not only at the initial phase of disease but also when it evolves towards the state classically defined as the castration-resistant PC (CRPC) condition. It has to be underlined, in fact, that most of CRPC is driven by the androgen axis despite castration levels of testosterone through a still not completely understood mechanism, including AR gene amplification and increased AR protein expression [7]. Androgen deprivation therapy (ADT) is the mainstay of the clinical management of advanced PC. In addition, the recent implementation of the so-called androgen-signaling inhibitors (ARSI), such as abiraterone acetate and enzalutamide, has thoroughly changed the therapeutic landscape of metastatic (m) CRPC [8].

AR is expressed in 70% of primary and metastatic BC, although with a certain degree of variability, according to the various BC subtypes, thus representing an appealing therapeutic target. In particular, AR is emerging as a potential weapon for managing ER-positive and triple-negative breast cancer (TNBC), which prognosis is still considerably poorer compared to the other BC subtypes due to the shortage of therapeutic options [9].

There is an unmet need for imaging biomarkers suitable for patients’ selection before enrollment with AR-targeted therapies and to dynamically investigate the eventual changes in AR expression during treatment in order to promptly detect the acquired resistance [10]. In this context, molecular imaging (MI) with positron emission tomography (PET) provides a unique opportunity to gain insight into tumor biology through imaging probes (i.e., radiopharmaceuticals or tracers) capable of binding specific tumor-associated biomarkers [11,12,13].

Several efforts have been made in order to develop radiopharmaceuticals suitable for the in vivo imaging of AR through PET technology. In this regard, fluoro-5α-dihydrotestosterone ([^18^F]-FDHT) has been synthesized and employed in preliminary clinical studies with promising results [14].

The aim of this systematic review is to provide a comprehensive overview of the first applications of [^18^F]-FDHT PET in patients affected by PC and BC, underlining the issues emerging from data analysis and shaping the future steps needed for its widespread implementation in clinical practice.

## 2. Materials and Methods

### 2.1. Search Strategy

A literature search until May 2023 was performed in the PubMed, Web of Science and Scopus databases in order to retrieve papers related to the topic, according to the Preferred Reporting Items for Systematic reviews and Meta-analyses (PRISMA) guidelines [15]. The terms used, with different combinations, were “dihydrotestosterone”, “DHT”, “[^18^F]-FDHT”, “breast cancer”, “prostate cancer” and “PET”. The following types of studies were considered: head-to-head comparative series, matched-pair studies, clinical trials, prospective studies and retrospective cohorts. Case reports, review papers, conference proceedings, editorial commentaries, interesting images and letters to the editor were excluded. Only studies published from January 2013 to May 2023, limited to humans and in the English language, were selected.

Two reviewers (L.F. and L.U.) conducted the literature search and independently appraised each article using a standard protocol and data extraction. The reference lists of the selected studies were carefully checked to identify any additional relevant literature.

From each study, the extracted data included the type of the study (prospective, retrospective, etc.); year and location of the study; sample size; oncological disease (e.g., breast or prostate cancer); primary endpoint and potential comparison with other tracers. Studies with incomplete technical or clinical data were considered ineligible.

### 2.2. Quality of the Selected Studies

The selected imaging studies were analyzed using a modified version of the Critical Appraisal Skills Programme (CASP) (https://casp-uk.net/aboutus, accessed on 30 May 2023) checklist for diagnostic test studies. A critical appraisal was performed by 2 reviewers (L.F. and L.E.), and discrepancies, if any, were solved by discussion among the authors.

## 3. Results

### 3.1. Analysis of the Evidence

**Figure 1:**
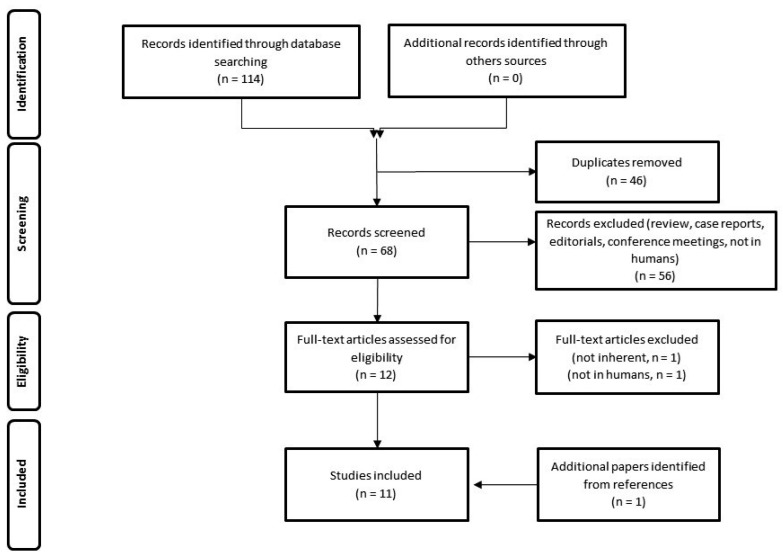
Schematic representation of PRISMA workflow for manuscript selection.

The resulting PRISMA search strategy is shown in Figure 1. From the systematic literature search, 11 papers [16,17,18,19,20,21,22,23,24,25,26], including an overall number of 321 patients, were finally selected. Table 1 summarizes the main findings of the selected manuscripts.

Seven of the eleven selected papers included 266 subjects (82.2%) affected by mCRPC and were mainly focused on the following thematic areas: (1) quantitation and feasibility studies (n = 4) mainly assessing the test/retest reproducibility of [^18^F]-FDHT PET, (2) a study evaluating the correlation between [^18^F]-FDHT uptake in lesions and AR expression using an immunohistochemical analysis and (3) studies (n = 2) focused on the prognostic role of [^18^F]-FDHT PET, with respect to [^18^F]-FDG PET, in mCRPC treated with AR-targeted therapies.

Four papers encompassed 55 (17.2%) patients affected by metastatic BC (mBC) and were subdivided as follows: (1) a feasibility study. (2) a correlative study between the [^18^F]-FDHT PET signal and AR expression in mBC tumor samples and (3) papers (n = 2) assessing the potential role of [^18^F]-FDHT PET for monitoring mBC patients submitted to experimental trials with AR-targeted treatments.

In 10 out of the 11 selected papers, a PET/CT scanner was used, while PET/MRI was employed in 1 report. A certain degree of heterogeneity was registered among the papers concerning the administered activity, modality (dynamic or static) or timing of acquisition. In particular, 10 out of 11 studies employed a fixed [^18^F]-FDHT activity ranging 200–350 MBq, while 1 study utilized an activity of 3 MBq/Kg. As concerns the acquisition protocol, two feasibility studies [18,19] utilized a p.i. 30 min dynamic scan followed by a whole body acquisition at 45 min, while the remaining papers employed a whole body scan at 30–60 min p.i.

**Figure 2:**
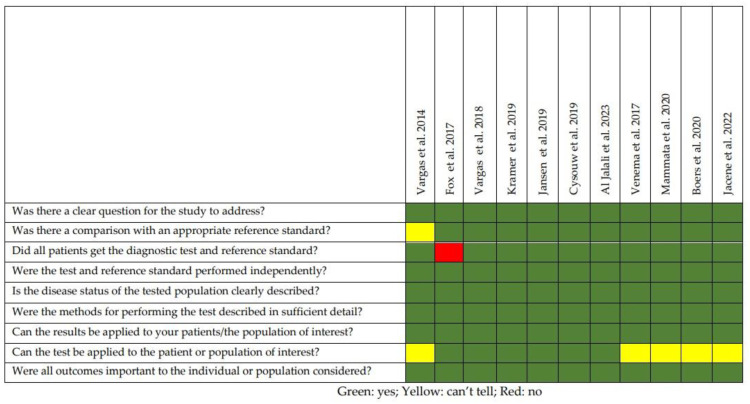
Quality appraisal of the selected articles using the CASP checklist for diagnostic studies [16,17,18,19,20,21,22,23,24,25,26].

The quality appraisal of the selected studies is represented in Figure 2. All the papers were prospective studies, and the majority of them utilized histology (i.e., histochemical analysis) as the reference standard. The main limitations of the studies were (1) the small sample sizes (only one study [17] included more than 50 patients); (2) all the selected papers were carried out in three countries (i.e., the USA, Austria and the Netherlands) and (3) two studies [19,24] included in their cohort patients collected from previously published reports.

The findings of the selected papers for each thematic area are described in the following sections.

### 3.2. Prostate Cancer

The implementation of [^18^F]-FDHT PET might represent a very useful tool in daily clinical practice, since ADT is a mainstay treatment in PC. However, the introduction of a new radiopharmaceutical agent has to be preceded by the publication of feasibility studies in the literature. Among those, a multicenter analysis of 252 patients performed by Jansen et al. [20] assessed the interpatient variability of the distribution of several different radiotracers used for PC imaging, including 27 [^18^F]-FDHT PET/CT scans. The [^18^F]-FDHT distribution was tendentially homogeneous, and the least interpatient variability was found in the liver. Thus, the authors concluded that a 3-cm VOI sphere placed in the liver should be used as a reference region to standardize interpatient analyses using [^18^F]-FDHT PET/CT. In another paper, a dynamic acquisition of [^18^F]-FDHT PET/CT was performed to evaluate the accuracy of different parameters that could be used to quantify the [^18^F]-FDHT uptake [19]. The SUV body weight (SUVbw), which is largely used in daily clinical practice, was not the best parameter in terms of accuracy (R2 = 0.70). Nevertheless, its accuracy was significantly improved by introducing a correction for sex and the hormone-binding globulin levels (R2 = 0.88), thus justifying its use in clinical practice. Similar to any new agent introduced in clinical practice, [^18^F]-FDHT PET imaging has to be validated and standardized according to the EANM Research Ltd. (EARL)-1 and EARL-2 guidelines [27,28]. The assessment of the reproducibility of [^18^F]-FDHT PET imaging was the aim of a paper published by Cysouw et al. [21], who performed a PET scan on two consecutive days on 14 mCRPC patients using different acquisition protocols. Interestingly, a reduction from the standard 3–4 to 1.5 min per bed position allowed a comparable detection rate of the scan, impairing only the evaluation of small lesions. This is a promising result if we consider that the cohort of patients performing [^18^F]-FDHT PET is usually represented by mCRPC patients, who could have difficulties in carrying out the whole duration of the PET scan due to pain related to bone metastases. This issue could be partially overwhelmed by the widespread use of modern long-axial field-of-view (LAFOV) PET tomographs, which provide superior diagnostic performances than traditional tomographs in shorter acquisition times [29]. Moreover, Cysouw et al. [21] also reported that SUVpeak could replace SUVmax, because it showed a variability <30% in lesions of at least 4.2 ml in volume without altering the counts, according to EARL-2 reconstruction. Hence, it could potentially be used for treatment response evaluations, which is another open question for PET imaging for PC patients [30,31].

Considering clinical papers, the article by Fox et al. [17] is surely worthy of mention. The authors prospectively performed both [^18^F]-FDHT and [^18^F]-FDG PET/CT on a cohort of 133 mCRPC patients. The comprehensive presence of 12 or more lesions detected using PET/CT scans and a median SUVmax > 7.6 on [^18^F]-FDG PET/CT resulted in strong negative prognostic factors in terms of a reduced overall survival (*p* < 0.001 and *p* = 0.07, respectively). Moreover, the lesions detected were divided into four subgroups according to their imaging results—in particular, considering the concordance of the uptake of the two different radiotracers. Interestingly, biopsies of lesions with [^18^F]-FDHT-positive and [^18^F]-FDG-negative PET scans (named the AR_1_Glyc_0_ group) had a predictive true-positive rate of 98%. This result means that detecting AR_1_Glyc_0_ lesions is highly specific for the localization of PC, and a confirmation biopsy is unnecessary. Conversely, patients presenting lesions belonging to the opposite group (named the AR_0_Glyc_1_ group) had the poorest prognosis, and the authors suggest performing a biopsy of these lesions to exclude a possible concomitant second neoplasm. In a previous study on 38 patients by Vargas et al. [16], a high uptake of [^18^F]-FDHT PET was also associated with a shorter OS (*p* = 0.02), suggesting that [^18^F]-FDHT PET could also have a prognostic relevance. Further studies are required to validate this preliminary evidence. Finally, in the paper by Jalali et al. [22], the authors compared [^18^F]-FDHT and [^68^Ga]-PSMA-11 PET/MRI in the primary staging of 10 patients with a new diagnosis of PC. While PSMA PET/MRI was superior to [^18^F]-FDHT PET/MRI for the primary PC diagnosis, [^18^F]-FDHT PET/MRI showed a stronger correlation between the uptake and AR expression during histopathology (r = 0.72). This evidence, if confirmed in larger cohorts, could be very useful for clinicians, providing an in vivo assessment of the AR status in PC patients. Indeed, AR expression and its alterations are very relevant for the selection of ADT agents and to detect the insurgency of resistance to these therapies.

### 3.3. Breast Cancer

**Figure 3:**
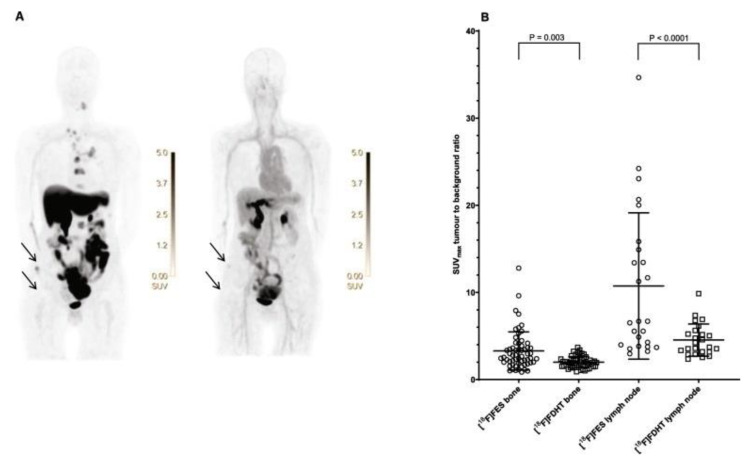
(**A**) An example of [^18^F]-FES (left side) vs. [^18^F] -FDHT (right side) in a female patient with mBC, demonstrating the greatest number of localizations on the bone and lymph nodes (arrows) detected by the former imaging approach. (**B**) Quantitative measures of tracers incorporated in the skeletal and nodal metastases (i.e., Cochran’s Q and McNemar tests were used to analyze differences in the SUVmax to the background ratios). Reprinted from [24] under a Creative Commons Attribution 4.0 International License (http://creativecommons.org/licenses/by/4.0/, accessed on 30 May 2023). No changes were made.

As previously mentioned, AR is present in 70–80% of breast carcinomas, which offers a potential new treatment strategy with AR-affecting drugs [32]. Because the ER is functionally and structurally highly comparable to the AR, responses to AR-targeting drugs may also rely on AR expression in the tumor. As demonstrated by some authors, in ER-positive BC, AR inhibits tumor proliferation [33,34]. The expression of AR and ER can be evaluated by using an immunohistochemical analysis, but it cannot be available at all sites of the disease, mainly in cases of multiple metastases, and therefore, it is essential to adopt alternatives. MI with [^18^F]-FDHT and [^18^F]-FES were tested in 13 patients by Venema et al. [23], showing a sensitivity of 91% and 100% and a specificity of 100% for the identification of AR and ER, respectively. Indeed, the authors demonstrated that [^18^F]-FDHT PET may be an interesting tool in selecting patients eligible for clinical trials with AR antagonists and to analyze the receptor occupancy of these drugs. Some years later, Mammatas et al. [24] analyzed the interobserver variability in interpreting [^18^F]-FDHT and [^18^F]-FES PET/CT in patients with BC (n = 10 patients). The authors found a high variability during the visual assessment but a good agreement during the quantitative analysis for [^18^F]-FDHT PET/CT, thus delineating the first approach for the correct interpretation of this imaging modality, as shown in Figure 3. Indeed, during the Bland–Altman analysis (performed to compare inter-reader reproducibility for [^18^F]-FDHT PET/CT images), the authors found an interclass correlation (ICC) of 23% by considering the visual assessment, but the value increased to 75% in the case of a quantitative analysis (such as SUVmax or SUVpeak).

In 2021, two papers were published about the utility of [^18^F]-FDHT PET/CT in predicting and evaluating the response to AR-directed therapy in BC patients [25,26]. Boers et al. [25] performed serial [^18^F]-FDHT PET/CT scans on 21 patients with AR-positive mBC receiving bicalutamide. The authors did not observe a clear association between changes in the [^18^F]-FDHT uptake and bicalutamide response. Indeed, patients with clinical benefits showed a nonsignificant reduction in tracer uptake compared to those with a progressive disease. However, when only ER-positive mBC patients were selected (n = 13), the change in [^18^F]-FDHT uptake was consistently higher for patients with a clinical benefit (n = 5) than those with a progressive disease (n = 8). Later, Jacene et al. [26] enrolled 11 ER-positive mBC patients who were studied with [^18^F]-FDHT PET/CT at the baseline and 6 and 12 weeks after starting a selective AR modulator called GTx-024 that is an oral nonsteroidal agent that specifically binds AR-promoting agonist activity, showing limited side effects. The median baseline FDHT-SUVmax was slightly higher for AR-positive tumors than AR-negative tumors. After 6 weeks and 12 weeks from the start of therapy, patients who experienced a clinical benefit showed a higher reduction in [^18^F]-FDHT uptake in the metastases than those who had a progression of the disease.

## 4. Discussion

In addition to sharing a strong dependence on sexual hormones, PC and BC are both considered dynamic malignancies, expressing shifting targets during their natural histories [35,36]. Some molecular determinants, such as AR or ER, might be relevant only in some phases of the disease due to the emergence of resistant clones. It is well known that the androgen axis has a crucial role in the management of advanced PC, and it is also emerging as an interesting potential target for BC. According to the preliminary clinical findings, PET with [18F]-FDHT may be a valuable method for quantifying and monitoring AR expression in vivo. In this respect, from the analysis of the selected papers, some considerations can be made. Indeed, some issues are still open, such as (1) the optimized protocol, (2) the prognostic role of [^18^F]-FDHT and (3) the complementary roles of [^18^F]-FDHT and radiolabeled PSMA. For the first point, the technological evolution of PET scanners will play an important role in the next few years; therefore, the synergism between technology and chemistry will be strategic [37,38]. For the second issue, few data are now available for coming to any final conclusions; indeed, the literature is also controversial about this point; however, [^18^F]-FDG still remains a consolidated point for the prognosis of patients with mCRPC. Finally, the low amount of data comparing radiolabeled PSMA ligands and [^18^F]-FDHT does not allow for some assumptions, although the targets of the two tracers are very different.

Lesional heterogeneity appears to play a significant part in the natural history of PC in light of the aforementioned, and physicians should always take this into consideration when choosing a course of treatment [35]. Nuclear medicine imaging could play a key role in the assessment of PC heterogeneity, particularly in the mCRPC setting, since several radiotracers with different molecular mechanisms are now available (i.e., PSMA ligands, [^18^F]-choline, [18F]-FACBC, [^18^F]-FDHT and [^18^F]-FDG) [39,40]. In this context, a deeper knowledge of the association between tracer uptake and radiogenomics is desirable for a precision medicine perspective [41].

The available data about [^18^F]-FDHT PET in BC patients are still limited in the literature (55 patients in total). The first experiences by Venema et al. [23] and Mammatas et al. [24] aimed to assess the accuracy of [^18^F]-FDHT in predicting the in vivo expression of AR in mBC and, later, to understand which method of interpretation would be reproducible. Later, Boers et al. [25] and Jacene et al. [26] tested the clinical efficacy of [^18^F]-FDHT PET in mBC patients treated with AR drugs, such as bicalutamide and GTx-04. However, the last two reports included a limited number of patients and, without a clear impact of the agent on the final outcome of patients, neither progression-free nor the overall survival. Therefore, the role of [^18^F]-FDHT PET in ER-positive mBC patients is still unknown, although it seems promising.

A few studies have correlated [^18^F]-FDHT uptake in lesions with the AR density determined by histochemistry. In PC patients, Jalali and coworkers [22] found a strong and significant correlation between [^18^F]-FDHT uptake in lesions (SUVmax/SUVbackground) and the AR optical density measured on tumor samples (r = 0.72), while ^68^Ga-PSMA-11, although demonstrating a correlation with PSMA expression in tissues, did not reach the threshold of statistical significance. As concerns mBC, Venema et al. [23] correlated [^18^F]-FES and [^18^F]-FDHT uptake (i.e., SUVmax) with the expression of ER and AR, respectively, measured in tumor samples: both the aforementioned tracers showed a significant correlation with the histochemical data, although it was greater between [^18^F]-FES and ER than in the case of [^18^F]-FDHT and AR (0.78 vs. 0.47). On the contrary, Jacene et al. [26] found a weak, not meaningful, correlation between the baseline [^18^F]-FDHT incorporation in lesions and AR-expression levels in mBC receiving AR-targeted therapy. Although these preliminary data warrant further confirmation, [^18^F]-FDHT seems to represent a reliable surrogate for AR expression in the case of PC, while its role in BC should be deepened by further investigations.

Notably, in order to implement simplified methods to quantify [^18^F]-FDHT on PET images as a surrogate biomaker of AR expression, rigorous kinetic modeling (KM) studies are mandatory. Kramer and colleagues [19] indicated that an irreversible two-tissue compartment model might be suitable for describing [^18^F]-FDHT kinetics in mCRPC lesions. However, these preliminary results were partially hampered by the small cohort of analyzed patients (n = 17) and by the fact that only a subset of six subjects underwent, aside from imaging, arterial blood sampling and dynamic ^15^O-H_2_O scans. In this respect, [^18^F]-FDHT KM remains an open question, and further investigations are auspicious.

Finally, comments are relative to the interpretation of [^18^F]-FDHT; indeed, both in mCRPC and mBC, SUVpeak seems a good semiquantitative parameter for the interpretation of PET images. However, additional findings are required to establish the best predictor for therapeutic assessment and prognosis.

## 5. Conclusions

In conclusion, [^18^F]-FDHT is an interesting agent for the in vivo evaluation of AR in mCRPC and mBC. Although its diagnostic, therapeutic and prognostic roles have yet to be fully explored, [^18^F]-FDHT holds the promise to be a key point for selected patients (i.e., ER-positive BC or in FDG-negative mCRPC). Further studies are needed to better define the role of [^18^F]-FDHT PET, alone or in combination with other tracers (i.e., [^18^F]-FDG/[^18^F]-FES), for patient selection and monitoring during AR-targeted therapy, especially in the case of mBC.

## Figures and Tables

**Table 1:** Main findings of the selected papers on the applications of [^18^F]-FDHT in prostate and breast cancer.

References	Location/Year	Study	N. of Patients	Setting	Median Age (Range)	Primary Endpoint	Device	Comment
Vargas et al. [16]	USA/2014	Prospective, observational	38	mCRPC	62.1 (43.1–76.0)	to compare CT, [^18^F]-FDHT, [^18^F]-FDG	PET/CT	The number of lesions detected on [^18^F]-FDG, [^18^F]-FDHT and CT has a prognostic impact on the OS, as well as the grade of [^18^F]-FDHT uptake in bone lesions.
Fox et al. [17]	USA/2017	Prospective, observational	133	mCRPC	68 (44–85)	To assess the prognostic role of [^18^F]-FDHT and [^18^F]-FDG in mCRPC submitted to ARSI	PET/CT	Four different phenotypes were identified. Lesions showing mismatch between [^18^F]-FDHT and [^18^F]-FDG (AR_0_Gly_1_ phenotype) had the poorest prognosis.
Vargas et al. [18]	USA/2018	Prospective, observational	27	mCRPC	----	To assess reproducibility and repeatability of [^18^F]-FDHT PET/CT	PET/CT	Uptake metrics (particularly the SUVmax mean peak) derived from [^18^F]-FDHT PET/CT showed high reproducibility and repeatability.
Kramer et al. [19]	USA/2019	Prospective, observational	17	mCRPC	69 (58–85)	To investigate if simplified metrics can be used to measure [^18^F]-FDHT uptake in lesions	PET/CT	SUV_BW_ corrected for sex hormone-binding globulin levels (SUV_SHBG_) may be used to quantify tracer uptake in lesions.
Jansen et al. [20]	the Netherlands/2019	Centralized analysis of a multicenter data	27	mCRPC	67 (64–69)	To assess the interpatient Variability of [^18^F]-FDHT uptake in healthy tissue	PET/CT	Low uptake variability was observed in all tissues, except the lungs. In particular, liver may be used as a reference region to characterize malignancies and standardize image interpretation
Cysouw et al. [21]	the Netherlands/2019	Prospective	14	mCRPC	----	To investigate how count statistics and reconstruction protocol affect lesion [^18^F]-FDHT PET quality image	PET/CT	Count reduction resulted in higher intrascan variability, regardless of the reconstruction method (EARL-1 or -2). However, the count statistics could be reduced without impacting lesions’ detectability.
Al Jalali et al. [22]	2023/Austria	Prospective, exploratory	10	mCRPC	60 (54–67)	To measure the correlation between [^18^F]-FDHT and [^68^Ga]-PSMA-11 uptake in PC and the expression of AR and PSMA at immunohistochemical analysis	PET/MRI	Although [^18^F]-FDHT was less sensitive than [^68^Ga]-PSMA-11 for the detection of the primary PC, a strong and significant correlation was found between the [^18^F]-FDHT imaging signal and AR density. [^18^F]-FDHT PET may be helpful to monitor the changes in AR expression during targeted therapies.
Venema et al. [23]	2017/Austria	Feasibility trial	13	mBC		To gauge the correlation between [^18^F]-FDHT and [^18^F]-FES imaging and the expression of AR and ER in tissues at at immunohistochemical analysis	PET/CT	A good correlation was found between the PET signal and AR density in tissues. The optimal cutoff for AR- positive lesions was an SUVmax of 1.94 for [^18^F]-FDHT PET.
Mammatas et al. [24]	the Netherlands/2020	Prospective	10	mBC	67 * (48–79)	To compare the visual and quantitative variability of [^18^F]-FES PET and [^18^F]-FDHT PET interpretation	PET/CT	[^18^F]-FDHT PET/CT showed lower visual agreement for a lesion’s detection than [^18^F]-FES but a good quantitative concordance.
Boers et al. [25]	the Netherlands/2021	Prospective	21	mBC (AR+/HER2-)	65 *	To investigate the usefulness of [^18^F]-FDHT PET for monitoring mBC treated with bicalutamide	PET/CT	Although a bicalutamide-induced [^18^F]-FDHT reduction was found on the follow-up PET/CT in mBC patients, this change was not predictive of the response.
Jacene et al. [26]	USA/2022	Prospective	11	mBC	59 (47–73)	To assess the role of [^18^F]-FDHT PET for monitoring mBC submitted to selective androgen receptor modulators (SARMs).	PET/CT	Patients showing a clinical benefit from SARMs showed a trend towards a progressive reduction in lesions’ [^18^F]-FDHT uptake on longitudinal PET/CT studies, with respect to subjects with a progressive disease.

mCRCPC: metastatic castration-resistant prostate cancer and mBC: metastatic breast cancer; *: mean age.
